# Photodynamic Priming Improves the Anti-Migratory Activity of Prostaglandin E Receptor 4 Antagonist in Cancer Cells In Vitro

**DOI:** 10.3390/cancers13215259

**Published:** 2021-10-20

**Authors:** Aaron J. Sorrin, Cindy Liu, Julia Cicalo, Jocelyn Reader, Daniel Najafali, Yuji Zhang, Dana M. Roque, Huang-Chiao Huang

**Affiliations:** 1 Fischell Department of Bioengineering, University of Maryland, College Park, MD 20742, USA; asorrin@umd.edu (A.J.S.); cliu1214@terpmail.umd.edu (C.L.); julia.cicalo.212@gmail.com (J.C.); danieln6@illinois.edu (D.N.); 2 Department of Obstetrics, Gynecology, and Reproductive Sciences, University of Maryland School of Medicine, Baltimore, MD 21201, USA; jreader@som.umaryland.edu (J.R.); droque@som.umaryland.edu (D.M.R.); 3 University of Maryland Greenebaum Comprehensive Cancer Center, Baltimore, MD 21201, USA; yuzhang@som.umaryland.edu; 4 Department of Epidemiology and Public Health, University of Maryland School of Medicine, Baltimore, MD 21201, USA

**Keywords:** prostaglandin inhibitor, photoimmunotherapy, antibody-drug conjugate, ovarian cancer, photodynamic therapy

## Abstract

**Simple Summary:**

Photodynamic priming is an emerging strategy that leverages subtherapeutic photochemistry for therapeutic benefits, often used as part of combination regimens. Our study aimed to couple photodynamically priming with antagonism of the prostaglandin E receptor 4, a therapeutic target linked to cancer-associated migration, invasion, angiogenesis, and immune evasion. Photodynamic priming and antagonism of the prostaglandin E receptor 4 independently attenuated OVCAR-5 ovarian cancer cell migration in a gap closure model, though their combination induced the most significant reductions. More potent combination effects were revealed when invasiveness was characterized using a transwell invasion model with CAOV3 ovarian cancer cells. Immunoblotting identified the epithelial growth factor receptor, cAMP-response element binding protein, and extracellular signal-regulated kinase 1/2 as potential mediators of these combinational effects. This work provides new evidence of a novel and clinically relevant combination strategy to address metastatic behavior, a major challenge in the treatment of cancer.

**Abstract:**

The combination of photodynamic agents and biological inhibitors is rapidly gaining attention for its promise and approval in treating advanced cancer. The activity of photodynamic treatment is mainly governed by the formation of reactive oxygen species upon light activation of photosensitizers. Exposure to reactive oxygen species above a threshold dose can induce cellular damage and cancer cell death, while the surviving cancer cells are “photodynamically primed”, or sensitized, to respond better to other drugs and biological treatments. Here, we report a new combination regimen of photodynamic priming (PDP) and prostaglandin E_2_ receptor 4 (EP4) inhibition that reduces the migration and invasion of two human ovarian cancer cell lines (OVCAR-5 and CAOV3) in vitro. PDP is achieved by red light activation of the FDA-approved photosensitizer, benzoporphyrin derivative (BPD), or a chemical conjugate composed of the BPD linked to cetuximab, an anti-epithelial growth factor receptor (EGFR) antibody. Immunoblotting data identify co-inhibition of EGFR, cAMP-response element binding protein (CREB), and extracellular signal-regulated kinase 1/2 (ERK1/2) as key in the signaling cascades modulated by the combination of EGFR-targeted PDP and EP4 inhibition. This study provides valuable insights into the development of a molecular-targeted photochemical strategy to improve the anti-metastatic effects of EP4 receptor antagonists.

## 1. Introduction

Photodynamic priming (PDP) is a powerful tool that leverages subtherapeutic photochemistry alone or in combination with chemotherapy or radiation therapy for cancer treatment. Its mechanism of action relies on the light activation of a photosensitizer molecule and subsequent generation of reactive oxygen species (ROS), resulting in biomolecule oxidation [1]. While the direct cell death brought about in this manner is associated with photodynamic therapy (PDT), PDP is achieved through sub-lethal effects [2]. These effects range widely from vascular modulation and chemo-sensitization to antitumor immune activation and remain under active investigation. Snyder et al. first showed that low dose photodynamic therapy could enhance macromolecule drug delivery through vascular permeabilization [3]. Several in vivo studies of rodent lung tumors revealed that low dose photodynamic therapy improved the distribution and delivery of liposomal chemotherapies [4,5,6]. Work by Debefve et al. further explored photochemical modulation of vasculature in the context of combination therapies [7,8], and later revealed that leukocytes play a major role in the vascular permeabilizing effects of photochemistry [9]. In addition to vascular modulation, PDP has also been shown to decrease tumor interstitial fluid pressure [10], attenuate chemotherapy selection pressure [11], overcome tumor desmoplasia by modulating tumor collagen content and extracellular matrix [12], enable chemotherapy dose-reduction [12,13], and enhance the cytotoxicity of radiation therapy [14]. A recent study also found that PDP using a triple-receptor-targeted formulation promoted antitumor immunity in pancreatic ductal adenocarcinoma spheroid cocultures with pancreatic cancer-associated fibroblasts and human peripheral blood mononuclear cells [15]. PDP upregulated the expression of antitumor immunogenic signals (Hsp60, Hsp70, CRT, and HMGB1) and induced activation of CD4+ and CD8+ T cells.

PDP-based combination regimens are currently under rapid development [11,12,13,14,15,16], and this study combines PDP with the inhibition of an emerging therapeutic target, the E-type prostanoid receptor 4 (EP4). EP4 is a G protein-coupled receptor that contributes to cancer progression and metastasis by promoting cancer cell invasion and migration, inducing tumor-associated angiogenesis, and attenuating the anti-cancer immune response [17,18,19]. EP4 is implicated in the onset and progression of numerous cancers including ovarian, lung, breast, uterine, colorectal, cervical, and prostate, among others [19,20,21]. A study by Spinella et al. showed that EP4 activation stimulates vascular endothelial growth factor (VEGF) production, cell migration, and matrix metalloproteinase activity in HEY human ovarian cancer cells [22]. Tonisen and colleagues also demonstrated that activation of EP4 was linked to invasive capabilities, invadopodia maturation, and matrix degradation in MDA-MB-231 breast cancer cells [18]. There are currently several clinical trials (NCT03658772, NCT03152370, NCT04344795, NCT04432857) investigating EP4 inhibitors for the treatment of colorectal cancer and other solid tumors, including endometrial and cervical cancers. These clinical trials are evaluating EP4 alone and in combination with chemotherapy, radiation therapy, or immunotherapy.

At the molecular level, EP4 has also been shown to intracellularly transactivate the epithelial growth factor receptor (EGFR) through the recruitment of β-arrestin and subsequent activation of membrane-bound Src in cancer cells [18,23]. EGFR signaling is linked to proliferation, migration, invasion, angiogenesis, and resistance to apoptosis in cancer cells [24,25]. Signaling pathways downstream of EP4 and EGFR are also overlapping; therefore, inhibiting EGFR alone may be insufficient. For example, preclinical work has shown that the simultaneous blockade of EGFR and EP4 outperforms the inhibition of EGFR alone in attenuating the tumorigenic cervical cancer cell signaling of mitogen-activated protein kinase (MAPK), cAMP-response element binding protein (CREB), protein kinase B (also called AKT), and glycogen synthase kinase (GSK) [26]. A recent study analyzed EP4 expression in ovarian tumor samples and found that EP4 was expressed in nearly 40% of the samples [27]. They also identified EP4 overexpression in several human ovarian cancer cell lines including OVCAR-3, CAOV3, SKOV3, and Kuramochi cells. EGFR is also overexpressed in 30–98% of epithelial ovarian malignancies [24]. This study develops a combination treatment of EGFR-targeted PDP and EP4 inhibition for ovarian cancer.

EGFR-targeted PDP is achieved by light activation of an antibody–photosensitizer conjugate using FDA-approved cetuximab (Cet) and a benzoporphyrin derivative (BPD) photosensitizer. The Cet-BPD conjugates used in this study are “cancer-activatable”, meaning that BPD molecules are quenched (inactivated) when conjugated to Cet, and can be un-quenched (activated) by cancer cells upon EGFR-mediated endocytosis and lysosomal proteolysis [28,29,30]. Preclinical studies showed that light activation of Cet-BPD is most effective when combined with chemotherapy for enhanced ovarian tumor burden reduction in vivo [28] and in vitro [31]. In 2020, a Cet-IRDye700 conjugate (also known as cetuximab saratolacan sodium, RM-1929, or ASP-1929) was approved for photoimmunotherapy of head and neck cancer in Japan [32,33,34]. The ASP-1929 photoimmunotherapy in combination with anti-PD1 therapy is currently under clinical investigation for patients with EGFR-expressing advanced solid tumors (NCT04305795). These studies suggest that the Cet-photosensitizer conjugate is an emerging therapeutic armamentarium against cancer, and its photodynamic efficacy may be further improved when combined with other treatment modalities.

Despite aggressive standard treatments consisting of platinum-taxane chemotherapy and cytoreductive surgery, roughly 80 percent of ovarian cancer patients will still develop recurrent disease [35]. Patients with resistant disease have a paucity of therapeutic options, and the need for novel treatment approaches is clear [36]. In this study, we evaluated the combination treatment of EGFR-targeted PDP and EP4 inhibition in the context of ovarian cancer migration, invasion, and metastasis-related cell signaling pathways linked to EP4 and EGFR. Gap closure and transwell invasion assays were used to characterize the anti-metastatic effects of BPD-based PDP, Cet-BPD-based PDP, and EP4 inhibition, alone and in combination, in two high-grade serous ovarian adenocarcinoma lines (OVCAR-5, CAOV3). Immunoblotting and enzyme-linked immunosorbent assays (ELISAs) are also conducted to further characterize molecular alterations induced by the treatments. This study provides new evidence that EGFR-targeted PDP coupled with EP4 inhibition attenuates cancer-promoting cell signaling and behaviors linked to metastasis in ovarian cancer cells.

## 2. Materials and Methods

### 2.1. Cell Culture

The high-grade serous ovarian adenocarcinoma cell lines, OVCAR-5 and CAOV3, were used in this study. OVCAR-5 cells were purchased from ATCC (Manasses, VA, USA) and the CAOV3 cell line was obtained from Dr. Giuliano Scarcelli (University of Maryland, College Park) who purchased the cells from ATCC (Manasses, VA, USA). Both cell lines were cultured in a 37 °C, 5% CO_2_ incubator. Cell lines were propagated for less than 40 passages, and cells were confirmed to be mycoplasma-free using the MycoAlert™ PLUS Mycoplasma Detection Kit (Lonza, Basel, Switzerland). RPMI-1640 medium with L-glutamine (Corning, Corning, MA, USA) containing 10% fetal bovine serum (FBS) (Gibco, Gaithersburg, MD), 100 U/mL penicillin, and 100 μg/mL streptomycin (Corning, Corning, MA, USA) were used to maintain OVCAR-5 cells. DMEM medium (Corning, Corning, MA, USA) supplemented with 10% FBS (Gibco, Gaithersburg, MD, USA) was used to maintain CAOV3 cells.

### 2.2. Gap Closure and Metabolic Activity Studies

For gap closure assays, OVCAR-5 cells were plated at 40,000 cells per well in 96-well plates, then treated with 2% serum-containing media containing DMSO (vehicle, < 0.5 %), BPD, Cet-BPD, AH23848, or a combination. After 24 h, cells were irradiated with a 690-nanometer laser (0.1 J/cm^2^, 10 mW/cm^2^, Modulight, Inc., Tampere, Finland). Irradiance was measured at the illuminated surface for each experiment, and black-walled wells were used for all studies to minimize reflected light. Monolayer cultures were scratched using an AutoScratch™ Wound Making Tool (Biotek, Winooski, VT, USA), and 5% serum-containing media was added to each well. AH23848 was re-added to the wells that had received prior AH23848 treatment. Imaging was performed with a Lionheart™ FX Automated Microscope (Biotek, Winooski, VT, USA), and image analysis was accomplished using Gen5 software (Biotek, Winooski, VT, USA). Gap closure percentage was calculated using the following equation: (initial gap area-final gap area)/initial gap area. Cellular metabolic activity studies, cell plating, and treatments were conducted the same as described in the gap closure protocol. An MTT (3-(4,5-dimethylthiazol-2-yl)-2,5-diphenyltetrazolium bromide) assay (Invitrogen, Waltham, MA, USA) was performed following the vendor’s protocol to assess relative metabolic activity for viability studies. For gap closure and metabolic activity studies, all experimental conditions were performed at least three times in triplicate.

### 2.3. Lysate Collection and Western Blotting

OVCAR-5 cells (1.1 × 10^6^) were plated in 35-millimeter cell culture dishes and treated with DMSO (vehicle), BPD, Cet-BPD, AH23848, or a combination in serum-free medium. After 24 h, dishes were irradiated with a 690-nanometer laser (0.1 J/cm^2^, 10 mW/cm^2^, Modulight, Inc., Tampere, Finland). After another 24 h, dishes were primed with 1 µM PGE_2_ and 50 ng/mL epithelial growth factor (EGF) (R&D, Minneapolis, MN, USA) in serum-free media for 10 min, then lysates were collected in RIPA buffer supplemented with 1% protease and phosphatase inhibitor cocktails (Thermo Fisher, Waltham, MA, USA). For CAOV3 lysate collections, 5 × 10^5^ cells were plated and PGE_2_ and EGF were not added. Western blotting was performed as previously described [37]. Proteins were detected using antibodies against EGFR (1:1000, Cell Signaling #4267), Phospho-EGFR (1:500, R&D MAB89671), ERK1 (1:1000, R&D AF1879), ERK2 (1:500, R&D MAB1230), and Phospho-Erk1/Erk2 (1:2000, R&D AF1018), CREB (1:1000, Cell Signaling #9104), Phospho-CREB (1:1000, Cell Signaling #9198), COX-2 (1:1000, Cell Signaling #12282), EP4 (1:500, Cayman #101775), MRP4 (1:500, Invitrogen #MA1-35681), and GAPDH (1:1000, Cell Signaling #2118). Membranes were imaged using the FluorChem E system (ProteinSimple, San Jose, CA, USA). For Western blotting, all experimental conditions were evaluated a minimum of four times. Signaling intensity of each protein marker was analyzed against GAPDH using ImageJ.

### 2.4. Photoimmunoconjugate Synthesis and Drugs

Photoimmunoconjugates Cet-BPD were synthesized at a ratio of ~4:1 (BPD:Cetuximab) by carbodiimide crosslinking of cetuximab to BPD, as described previously [31]. Total protein was quantified using a BCA assay and the BPD concentration was characterized using UV–Vis spectroscopy for photoimmunoconjugate characterization. AH23848 and PGE_2_ were obtained from Cayman Chemical (Ann Arbor, MI, USA). EGF was obtained from R&D Systems (Minneapolis, MN, USA).

### 2.5. Extraction Methods to Quantify Photosensitizer Uptake in Cells

OVCAR-5 cells were plated in 35-millimeter dishes at 1.1 × 10^6^ cells per dish, then treated with BPD or Cet-BPD. After 24 h, cells were lysed in RIPA buffer supplemented with 1% protease and phosphatase inhibitor cocktails (Thermo Fisher, Waltham, MA, USA) and then BPD fluorescence signal was measured using a plate reader (Synergy Neo2; Biotek, Winooski, VT, USA; Ex/Em: 435 nm/700 nm). Intracellular BPD concentrations were quantified by correlating fluorescence to a standard curve, then normalized to total protein level (grams) as determined using a BCA assay. All experimental conditions were performed at least three times in duplicate.

### 2.6. Transwell Invasion Assay and PGE_2_ ELISA

CAOV3 cells were plated in 35-millimeter dishes at 150,000 cells per dish (for invasion assay) or 500,000 cells per dish (for PGE_2_ ELISA). Cells were treated with vehicle (DMSO), BPD, Cet-BPD, AH23848, or a combination regimen in serum-free media for 24 h, then irradiated at 690 nm (0.1 J/cm^2^, 10 mW/cm^2^, Modulight, Inc., Tampere, Finland). For invasion assays, dishes were trypsinized and plated at 25,000 cells per well in the CultreCoat^®^ 96-Well Medium BME Cell Invasion Assay (R&D, Minneapolis, MN, USA). The remainder of the assay was conducted as per the manufacturer’s instructions. For the PGE_2_ ELISA, cell culture supernatants were collected 1 and 4 h after light-activation, then supernatants were assayed for PGE_2_ using the Prostaglandin E_2_ ELISA Kit (514010, Cayman Chemical, Ann Arbor, MI, USA). For transwell assays and ELISAs, all conditions were performed a minimum of three times in triplicate.

### 2.7. Statistical Analysis

Statistical analysis was conducted using GraphPad PRISM version 9.0.2 for MacOS, and ImageJ was used to quantify immunoblotting bands. Data for gap closure, transwell invasion, Western blotting, ELISA, and MTT studies were analyzed using one-way ANOVA followed by a post hoc Tukey’s test. F-tests were used to quantify changes in variance between groups. A value of *p* ≤ 0.05 was considered statistically significant.

## 3. Results

### 3.1. Combination of BPD-Based PDP and EP4 Inhibitor (AH23848) Decreases Ovarian Cancer Cell Migration and Invasion

**Figure 1:**
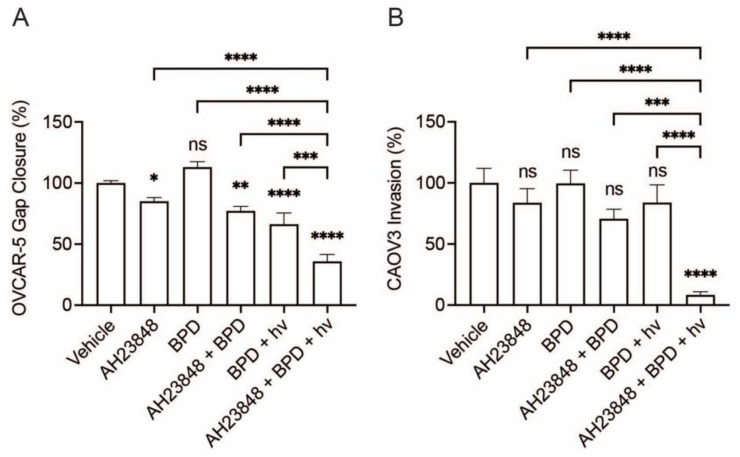
Anti-migratory effects of BPD-based PDP, EP4 inhibitor (AH23848), and their combination were evaluated in (**A**) a gap closure assay using OVCAR-5 cells and (**B**) the transwell invasion assays using CAOV3 cells. All data are normalized to the vehicle (DMSO) control, and statistical analysis was performed using a one-way ANOVA and post hoc Tukey’s test. Error bars represent the standard error of the mean. * *p* ≤ 0.05; ** *p* ≤ 0.01; *** *p* ≤ 0.001; **** *p* ≤ 0.0001; ns: nonsignificant.

To assess the effects of combination therapy with BPD-based PDP and AH23848 on human ovarian cancer cell migration, we performed the gap closure and transwell cell invasion assays using OVCAR-5 and CAOV3 cell lines (Figure 1). The concentrations of BPD and AH23848 were fixed at 0.5 and 40 µM, respectively, to maintain sublethal dosing (< 15% reduction in metabolic activity) upon light aviation (Supplementary Figure S1). The OVCAR-5 cells incubated with BPD without light activation showed no significant change in gap closure compared to the vehicle control (Figure 1A). When the OVCAR-5 cells were exposed to AH23848 with or without BPD, the cells migrated ~18% slower than that of the vehicle control (*p* < 0.05), demonstrating sensitivity to EP4 inhibition. PDP using light-activated BPD decreased gap closure by ~33% (*p* ≤ 0.0001). When the OVCAR-5 cells were treated with both BPD-based PDP and AH23848, there was a ~65% reduction in gap closure, which is significantly lower than that of the vehicle control and monotherapies (*p* ≤ 0.001). A stronger combination effect was observed in the CAOV3 cells using the transwell cell invasion assay (Figure 1B). Treatments with AH23848, BPD, their combination, or BPD-based PDP did not significantly alter CAOV3 migration compared to the vehicle control (*p* > 0.05). In contrast, the combination of BPD-based PDP and AH23848 greatly reduced the invasion of the CAOV3 cells by ~92%, and this was significantly lower than all the control groups (*p* ≤ 0.0001). Our data show that the combination of AH23848 and BPD-based PDP reduced the migration and invasion of two ovarian cancer cell lines in vitro.

### 3.2. BPD-Based PDP Combined with EP4 Inhibition Does Not Attenuate Cell Signaling Pathways Linked to EP4 and EGFR

**Figure 2:**
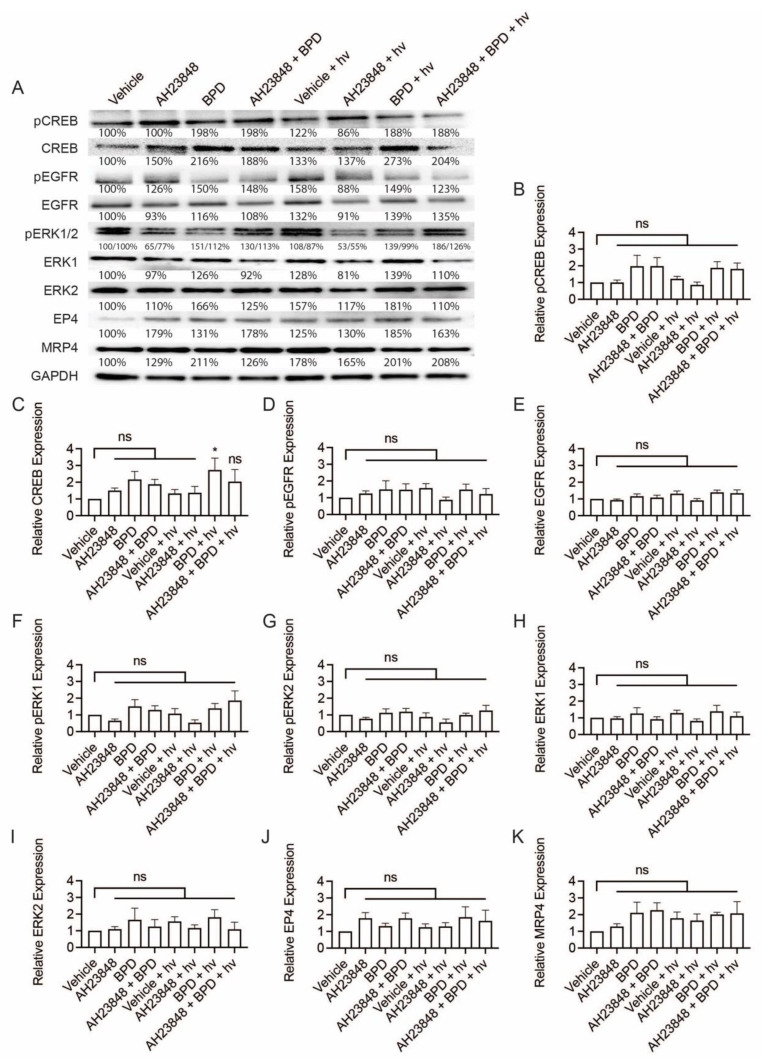
Western blot analysis of p-CREB, CREB, p-EGFR, EGFR, p-ERK1/2, ERK1, ERK2, EP4, and MRP4 in OVCAR-5 cells. Cells were treated with the indicated agents for 24 h, then light-activated (0.1 J/cm^2^, 10 mW/cm^2^) or maintained in dark conditions. After 24 h, cells were agonized with EGF (50 ng/mL) and PGE2 (1 µM) for 10 min, then whole extracts were collected and analyzed using Western blot. (**A**) Representative Western blot images and (**B**–**K**) relative densitometric bar graphs of phosphorylated and total proteins were shown. Results are normalized to the vehicle control group. Statistical analysis was performed using a one-way ANOVA and post hoc Tukey’s test. Percentages below each band represent the average change in intensity relative to the vehicle control across all experiments. For pERK1 and pERK2 bands, the first number corresponds to pERK1, and the second number corresponds to pERK2. Error bars represent the standard error of the mean. * *p* ≤ 0.05; ns: nonsignificant. Original western blot images (Supplementary Figure S4).

Considering the tumorigenic role of EP4 signaling and EP4-EGFR crosstalk, we investigated the expression of pCREB, CREB, pEGFR, EGFR, p-ERK1/2, ERK1, ERK2, EP4, and MRP4 in OVCAR-5 cancer cells following the combination treatment of BPD-based PDP and AH23848 (Figure 2A). Briefly, OVCAR-5 cells were treated with AH23848, BPD, or their combination for 24 h followed by light activation (hv, 0.1 J/cm^2^, 10 mW/cm^2^). Cells lysates were then collected at 24 h after treatment and used for Western blot analyses. Dark controls were included for comparison. Cells treated with BPD, with and without light and AH23848, showed an average of a two-fold increase in CREB expression. However, further analysis suggested that changes in pCREB expression was not statistically significant (Figure 2B). The only statistically significant change observed was an increase in CREB expression following BPD-based PDP (*p* ≤ 0.05, Figure 2C). The expression of p-EGFR, EGFR, p-ERK1/2, ERK1, ERK2, EP4, and MRP4 in OVCAR-5 cells did not change significantly following any treatment compared to the vehicle control (Figure 2D–K). Our data suggested that the combination of BPD-based PDP and AH23848 has minimal impact on the EGFR and EP4 signaling pathways. These findings motivated us to further investigate PDP using EGFR-targeted Cet-BPD in combination with AH23848 in subsequent studies to achieve the co-inhibition of EGFR and EP4.

### 3.3. Cet-BPD-PDP and BPD-PDP Have Similar Effects on Gap Closure When Compared at Equivalent Intracellular Photosensitizer Concentrations

**Figure 3:**
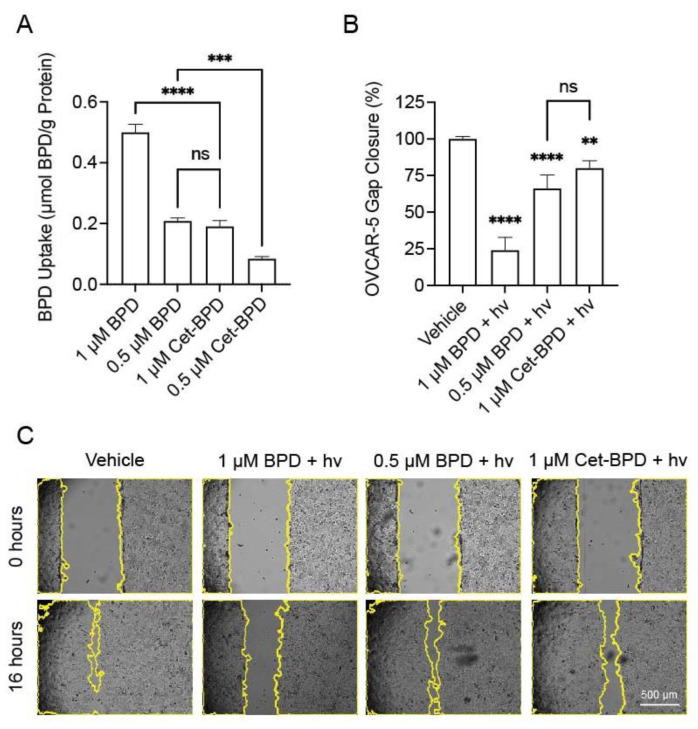
Conjugation of BPD to cetuximab impacts uptake and gap closure effects in ovarian cancer cells. OVCAR-5 cells were plated in 96-well plates, treated with the indicated BPD or Cet-BPD doses for 24 h, then (**A**) agents were extracted from cells to quantify cellular photosensitizer uptake, or (**B**) cells were light-activated at 690 nm and scratched for gap-closure analysis. Representative gap closure images are included (**C**). Statistical analysis was performed using a one-way ANOVA and post hoc Tukey’s test. Error bars represent the standard error of the mean. ** *p* ≤ 0.01; *** *p* ≤ 0.001; **** *p* ≤ 0.0001; ns: nonsignificant.

The uptake of Cet-BPD and effects on metastasis-related phenotype were assessed in the OVCAR-5 cells (Figure 3). Extraction studies showed that the intracellular accumulation of Cet-BPD was ~2.5-fold lower than that of free BPD (Figure 3A, *p* ≤ 0.001). At a fixed photosensitizer incubation concentration of 1 μM, a 24-h incubation of free BPD resulted in an intracellular photosensitizer concentration of ~0.5 μmoles of BPD per grams (g) of protein, compared to ~0.2 μmoles of BPD per grams (g) of protein for the OVCAR-5 cells treated with Cet-BPD. When the photosensitizer incubation concentration was fixed at 0.5 μM, the uptake concentrations for free BPD and Cet-BPD were ~0.2 and ~0.1 μmol BPD/g protein, respectively. Interestingly, treatment with 1 μM Cet-BPD and 0.5 μM BPD led to statistically equivalent amounts of photosensitizer uptake. As a result, these doses were further compared in gap closure assays (Figure 3B,C). PDP with light (hv) activation of 1 and 0.5 μM BPD reduced the gap closure by ~75 and ~35%, respectively, compared to the vehicle control (*p* ≤ 0.0001). Similarly, light (hv) activation of 1 μM Cet-BPD reduced the gap closure by ~24% (*p* ≤ 0.0001) compared to the control. Further analysis showed that there is no statistically significant difference between the anti-migratory effects of PDP using 0.5 μM BPD or 1 μM Cet-BPD (e.g., photosensitizer doses that result in equivalent intracellular concentrations). The 1 μM Cet-BPD treatment was, therefore, selected for use in subsequent studies to evaluate combination effects with EP4 inhibition.

### 3.4. Cet-BPD-Based PDP Combined with EP4 Inhibition Attenuates Migration, Invasion, and Cell Signaling Linked to EP4 and EGFR

**Figure 4:**
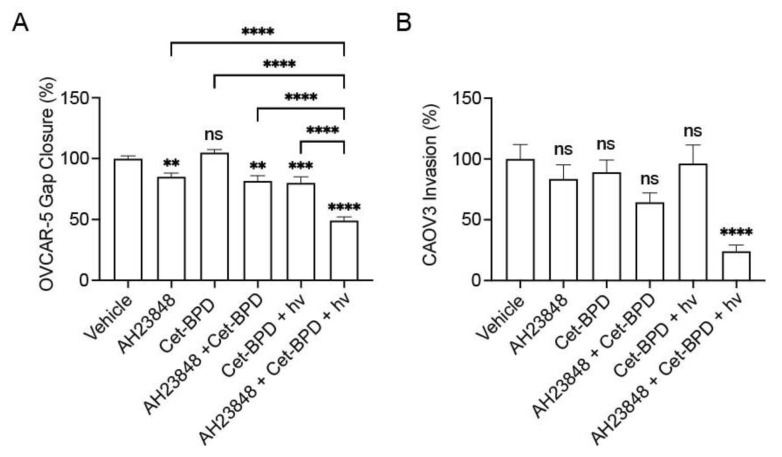
Investigation of anti-metastatic effects of Cet-BPD-based PDP combined with EP4 inhibition (AH23848). Treatments are evaluated in gap closure assays using OVCAR-5 cells (**A**) and transwell invasion assays using CAOV3 cells (**B**). All data are normalized to the vehicle (DMSO) control, and statistical analysis was performed using a one-way ANOVA and post hoc Tukey’s test. Error bars represent the standard error of the mean. ** *p* ≤ 0.01; *** *p* ≤ 0.001; **** *p* ≤ 0.0001; ns: nonsignificant.

In Figure 1, we showed that BPD-based PDP enhances the anti-migratory activity of EP4 inhibitors in ovarian cancer cells (Figure 1). We next investigated if Cet-BPD-based PDP combined with AH23848 also inhibited migration as measured using a gap closure assay or invasion as measured using a transwell assay (Figure 4). Cet-BPD alone at 1 μM did not induce significant alterations in gap closure (*p* > 0.05) in the OVCAR-5 cells; however, Cet-BPD-based PDP induced a 20% reduction in migration relative to the control (Figure 4A). EP4 inhibition using 40 μM AH23848 with and without Cet-BPD (1 μM) reduced gap closure by approximately 15% compared to the control OVCAR-5 cells. A combination of Cet-BPD-based PDP and AH23848 (40 μM) significantly reduced the OVCAR-5 gap closure by up to 50% of all the control groups (*p* < 0.0001). Similar effects were observed when combining Cet-BPD-based PDP with a lower concentration of AH23848 at 20 μM (Supplemental Figure S2). These data show that Cet-BPD-based PDP combined with AH23848 significantly inhibited ovarian cancer cell migration compared to both Cet-BPD-based PDP or AH23848 alone, demonstrating the superior potency of this combination regimen. Next, transwell invasion assays were conducted using the same treatment groups to characterize effects on CAOV3 invasion (Figure 4B). Treatment with AH23848 alone, Cet-BPD alone, a combination of Cet-BPD with an EP4 inhibitor, Cet-BPD-based PDP (Cet-BPD + hv), all resulted in a modest (4–30%) (but statistically insignificant) reduction in invasion. Only when the CAOV3 cells were treated with the combination of Cet-BPD-based PDP and AH23848 was a significantly reduction in the CAOV3 cell invasion (*p* ≤ 0.0001) by 76% observed, demonstrating a potent combination effect in the context of cell invasion.

**Figure 5:**
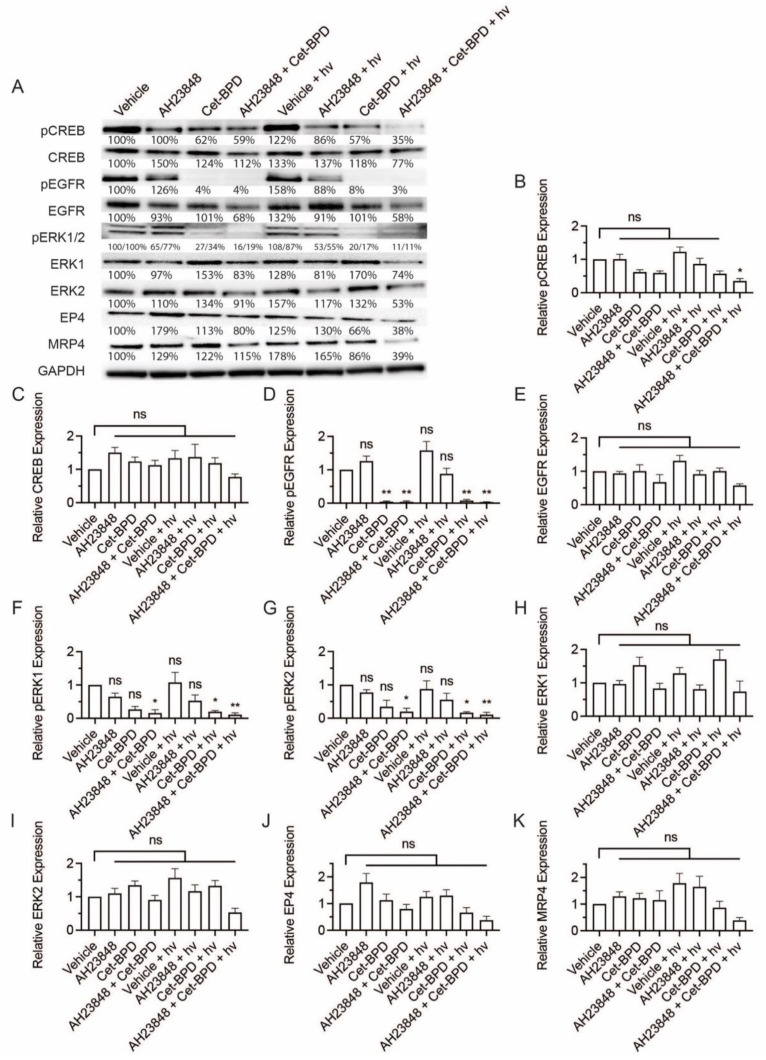
Western blot analysis of p-CREB, CREB, p-EGFR, EGFR, p-ERK1/2, ERK1, ERK2, EP4, and MRP4 in OVCAR-5 cells. Cells were treated with the indicated agents for 24 h, then light-activated (0.1 J/cm^2^, 10 mW/cm^2^) or maintained in dark conditions. After 24 h, cells were agonized with EGF (50 ng/mL) and PGE_2_ (1 µM) for 10 min, then whole extracts were collected and analyzed using Western blot. (**A**) Representative Western blot images and (**B**–**K**) relative densitometric bar graphs of phosphorylated and total proteins were shown. Results are normalized to the vehicle control group. Statistical analysis was performed using a one-way ANOVA and post hoc Tukey’s test. Percentages below each band represent the average change in intensity relative to the vehicle control across all experiments. For pERK1 and pERK2 bands, the first number corresponds to pERK1, and the second number corresponds to pERK2. Error bars represent the standard error of the mean. * *p* ≤ 0.05; ** *p* ≤ 0.01; ns: nonsignificant. Original western blot images (Supplementary Figure S5).

**Figure 6:**
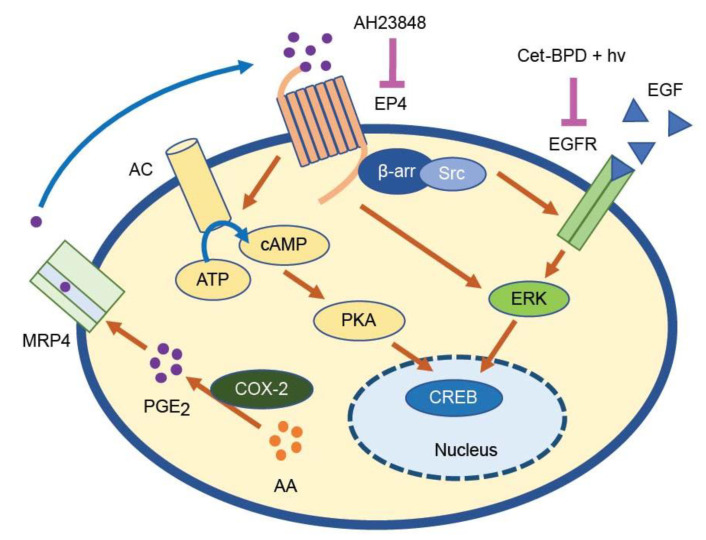
Proposed relationship between the combination treatment (Cet-BPD-based PDP and AH23848) and EGFR-EP4 signal transduction pathways. Arachidonic acid is converted to PGE_2_ by COX-1, COX-2, and PGE synthase [38]. PGE_2_ is exported from the cell via multiple drug resistance-associated protein 4 (MRP4), where it can bind to the G-protein coupled receptors, EP1–4 [39]. EP4 is coupled to the G protein alpha stimulator (Gs), which activates adenylyl cyclase. Adenylyl cyclase converts adenosine triphosphate (ATP) to cyclic adenosine monophosphate (cAMP), which subsequently activates Protein Kinase A (PKA). When PKA is activated, its catalytic subunits translocate into the nucleus and activate CREB, a transcription factor with complex roles in cancer [40]. EGFR can be activated extracellularly via EGF binding and intracellularly via the EP4/β-arrestin (β-arr)/Src complex [18]. Activated EGFR causes a variety of downstream effects including ERK phosphorylation, which is linked to CREB activation. EP4 has also been shown to induce ERK activation independently of EGFR [41]. The Cet-BPD and EP4 inhibitor combination regimen is designed to simultaneously abrogate EGFR and EP4 signaling to block tumorigenic crosstalk along with overlapping signaling pathways. Abbreviations: AA (arachidonic acid); COX2 (cyclooxygenase-2); PGE2 (prostaglandin E2); MRP4 (multidrug resistance-associated protein 4); EP4 (prostaglandin E2 receptor 4); ATP (adenosine triphosphate); cAMP (cyclic adenosine monophosphate); PKA (protein kinase A); CREB (cyclic AMP response element-binding protein); ERK1/2 (extracellular signal-regulated kinases 1/2); β-arr (β-Arrestin); EGFR (epidermal growth factor receptor); EGF (epidermal growth factor); BPD (benzoporphyrin derivative); Cet (cetuximab).

Cell signaling pathways associated with the activation of EGFR and EP4 were next evaluated using immunoblotting of the OVCAR-5 cells following treatment with Cet-BPD-based PDP and AH23848, alone and in combination (Figure 5). Representative images are displayed in Figure 5A. Cet-BPD combined with AH23848 attenuated pCREB signaling to 60% of the control and adding light further reduced pCREB activation to 35% (*p* ≤ 0.05). All the groups with Cet-BPD, regardless of the inclusion of AH23848, showed significant reductions in EGFR phosphorylation (*p* ≤ 0.01). In pERK1 and pERK2 signaling, the combination of Cet-BPD and AH23848 reduced signaling drastically by over 80% (*p* ≤ 0.05). Cet-BPD-based PDP combined with AH23848 further reduced pERK1/2 by 90% (*p* ≤ 0.01). None of the changes to total protein in CREB, EGFR, ERK1, ERK2, EP4, or MRP4 reached statistical significance. The molecular effects of co-inhibition of EP4 and EGFR using AH23848 and Cet-BPD are summarized in Figure 6.

## 4. Discussion

In this study, we show that PDP significantly attenuates gap closure in OVCAR-5 cells. This is consistent with previous work by Jiang et al., who showed, using an invasion assay, that Photofrin^®^-based subcytotoxic photochemistry inhibited glioblastoma transit through a Matrigel membrane [42]. Yang et al. demonstrated that sub-lethal photodynamic therapy (10–20% cell killing) using 5-Aminolevulinic acid (5-ALA) induced significant decreases in the migration and invasion of multiple head and neck cancer cell lines [43]. In our study, PDP was evaluated using two platforms: freeform BPD, as well as the EGFR-targeted Cet-BPD conjugate. The porphyrin-based BPD was selected due to its Food and Drug Administration approval status and because most earlier PDP studies use BPD or other porphyrin-based photosensitizers. However, Overchuk et al. recently used a bacteriochlorin-based photosensitizer to achieve PDP [12]. More work is warranted to characterize differences in priming effects between photosensitizers, or if a combinational approach of multiple photosensitizers may be beneficial. Figure 3 revealed that approximately two times more BPD is internalized compared to Cet-BPD. However, when compared at equal intracellular concentrations, BPD and Cet-BPD had similar effects on gap closure. Cetuximab-photosensitizer conjugates are rapidly gaining traction in the clinical sphere. In September 2020, Japan approved a Cet-IR700 construct, Akalux^®^, for the treatment of unresectable locally advanced or recurrent head and neck cancer. A Cet-IR700 construct (ASP-1929) is also under evaluation in two actively accruing clinical trials both alone (NCT03769506) and in combination with anti-PD1 therapy (NCT04305795).

We further demonstrated, for the first time, that incorporating EP4 inhibition into a PDP treatment led to additional reductions in migration along with a drastic attenuation of cell invasion (Figure 1 and Figure 4). To inhibit EP4, we used AH23848, which was first reported by Coleman et al. to antagonize EP4 in 1994 [44]. While AH23848 is commonly used in vitro, numerous EP4 antagonists have been developed with higher selectivity (CJ-023,423 (grapiprant), L-161982, ONO AE3-208, etc.) [23]. In this study, AH23848 was used as a model drug to validate the combination effect of EP4 inhibition with EGFR-targeted PDP. In addition to lowering migration and invasion, AH23848 combined with PDP also demonstrated a substantial increase in treatment consistency compared to PDP alone. To quantify this, F-tests were performed to compare the variances of BPD-based PDP and Cet-BPD-based PDP with and without AH23848. The analyses revealed statistically significant decreases in variance (alpha = 0.05) when AH23848 was added to both BPD-based PDP and Cet-BPD-based PDP in both migration studies and invasion studies (Figure 1 and Figure 4). The potent combination effects demonstrated here motivate future work using newer EP4 antagonists that are currently in clinical trials, including grapiprant, TPST-1495, and AN0025 (previously E7046).

Previous work supports our findings that EP4 plays a fundamental role in cancer progression. In murine breast cancer models, EP4 antagonism has been shown to reduce primary tumor growth, stem cell-like functions, tumor-associated angiogenesis and lymphangiogenesis, and metastasis to the lymph nodes and lungs [45,46]. Xu et al. showed in PC-3 prostate cancer cells that EP4 antagonism (or EP4 siRNA) attenuates the PGE2-mediated expression of matrix metalloproteinases, nuclear factor-κB ligand, and runt-related transcription factor 2, which promote cell growth and metastasis in multiple cancers [47]. We also recently showed that EP4 antagonism significantly reduced SK-UT-1 (leiomyosarcoma) cell migration and sensitized cells to docetaxel (IC_50_ decreased from 1.47 to 0.46 nM) [48]. Additionally, the intracellular crosstalk between EP4 and EGFR via EP4/β-arrestin/Src is well characterized [18,23,49]. In light of this, the co-inhibition of both receptors is a promising prospect that has been studied previously in cervical cancer cells by Parida et al. [26]. Cells were stimulated with PGE_2_ and treated with either an EP4 inhibitor (GW627368X), an EGFR monoclonal antibody, or both, then screened via Western blot for MAPK, CREB, AKT, and GSK phosphorylation. While the monotherapies produced potent downregulation in phosphorylation, the simultaneous blockade of EGFR and EP4 led to further reductions for multiple targets. This supports the notion that silencing compensatory signaling pathways can enhance treatment effects. Our study expands on this concept by coupling the co-inhibition of EP4 and EGFR with PDP.

While BPD-based PDP combined with EP4 inhibition (Figure 2) did not block the tumorigenic signaling of CREB, EGFR, ERK1, or ERK2, the EGFR-EP4 co-inhibition strategy resulted in potent downregulations (Figure 5). We show that phosphorylated EGFR is decreased in the presence of Cet-BPD, regardless of the addition of AH23848 or light-activation (Figure 5C). This is consistent with previous work by Abu-Yousif et al., who showed that Cet-BPD blocked EGFR phosphorylation in EGF-primed OVCAR-5 cells with and without light-activation [50]. The same study also looked at p-MAPK/ERK signaling and their Cet-BPD treatment only inhibited ERK phosphorylation when light-activated. Similarly, our study also showed that Cet-BPD-based PDP attenuates ERK phosphorylation. Unlike their study, we found that Cet-BPD without light activation also blocked ERK phosphorylation, though it was not a statistically significant decrease relative to the vehicle control. This difference can likely be attributed to the higher Cet concentration used in our study (~250 nM vs. 37 nM). Work by Cherukuri et al. showed that PGE_2_ stimulates ERK and CREB phosphorylation in colon cancer cells, and this can be blocked using a selective EP4 inhibitor (L-161,982) [51]. Our study is partially consistent with this, as we show that EP4 inhibition using AH23848 attenuates ERK1 and ERK2 phosphorylation to ~60% of the vehicle control. Unlike their study, inhibition of EP4 alone did not block the phosphorylation of CREB, likely due to the presence of the EGF-mediated stimulation of EGFR. In fact, multiple studies have linked EGFR to CREB activation [52,53], which is consistent with our data showing that Cet-BPD alone can modestly attenuate CREB phosphorylation with or without light activation. Importantly, the only treatment to induce significant reductions in CREB phosphorylation was Cet-BPD-based PDP combined with AH23848, demonstrating potent combination effects (Figure 5B). While previous work suggests that photochemistry upregulates both PGE_2_ and COX-2 [54,55,56], we did not observe the stimulation of either in our experiments (Supplemental Figure S3). Work by Ferrario et al. shows that the effects of photochemistry on COX-2 and PGE_2_ are highly dependent on dosage [54]; this likely explains our results because the light dose used (0.1 J/cm^2^) was relatively low. In contrast, we observed a notable downregulation of PGE_2_ in the supernatants following all treatments, particularly at 4 h.

PDP is a promising modality that leverages subtherapeutic (below the usually delivered dose) photodynamic therapy alone or as part of a combination regimen for cancer treatment. We envision that PDP can be in incorporated into the clinic in two ways. First, PDP can be achieved in the tissues surrounding photodynamic therapy-treated areas, as shown by Vincent et al. [57]. While these surrounding tissues receive subtherapeutic photochemistry, the PDP effects may be leveraged to enhance overall outcomes either through activating antitumor immunity or increasing the accumulation of another agent. Second, PDP can be used in the clinic as a tool to precisely enhance the delivery and selectivity of chemotherapy to tumors. Wang et al. showed that while high fluence PDT (30, 50 J/cm^2^) induced vascular occlusion in rodent mesothelioma xenograft tumors, using an intermediate fluence (10 J/cm^2^) improved FITC-Dextran leakage in tumors but not normal tissues [58]. Importantly, using a lower fluence (5 J/cm^2^) did not improve tumor uptake of FITC-Dextran, highlighting the importance of careful light dose selection for achieving the desired effects. They further demonstrated that photochemistry at 10 J/cm^2^ combined with liposomal cisplatin outperformed the monotherapies in inhibiting tumor growth. The use of PDP for selective chemotherapy delivery to tumors is, therefore, a promising avenue through which we envision PDP may be incorporated into a clinical setting.

It is important to acknowledge several limitations of our study. Transwell migration assays are subject to irregular migration and reproducibility issues, and in gap closure assays there can be mechanical damage to the cells and plate surface caused by the scratch as well as general reproducibility issues [59]. Both transwell and scratch assays are also performed on cells plated in two-dimensional monolayers, which do not replicate the three-dimensional structure of tumors. Future studies can be performed using 3D cultures and co-cultures that mimic the collective cell migration of cancer cells due to tumor cell-specific intercellular connections, tissue scaffold environment interactions, and interactions with tumor-associated cells.

## 5. Conclusions

This study demonstrates that PDP improves the anti-migratory activity of a prostaglandin E receptor 4 antagonist in ovarian cancer cells. We confirm this using two models of metastatic behavior (gap closure and invasion assays), two ovarian cancer cell lines (OVCAR-5 and CAOV3), and two photosensitizer formulations (non-targeted BPD and EGFR-targeted Cet-BPD). Molecular analysis indicates that EGFR, ERK1/2, and CREB signaling are implicated in these treatment outcomes. Based on these promising functional and mechanistic in vitro assays, further experiments to verify in vivo efficacy are warranted. It is also important to mention that cell migration and invasion are two parts of the complex, multi-step metastatic cascade. This cascade involves proteolytic remodeling of the basement membrane, cross-talk with stromal cells, invasion, transport along vascular and lymphatic routes, extravasation, and formation of metastatic niches [60]. Therefore, further studies to investigate the role of the PDP-EP4 combination in the context of these other steps would elucidate the holistic impact of the treatment in regulating cancer metastasis. Importantly, in addition to overexpression in cancer cells, EP4 is also expressed in various immune cells (i.e., macrophages, T cells, NK cells, and B cells), and PGE_2_-EP4 signaling plays a major role in evasion of the antitumor immune response [19,61,62]. Future in vivo work to study the PDP-EP4 inhibitor combination regimen should therefore evaluate anti-metastatic effects as well as the modulation of the antitumor immune response.

## Data Availability

All the data relative to this study are presented in the manuscript.

## Supplementary Materials

The following are available online at https://www.mdpi.com/article/10.3390/cancers13215259/s1, Figure S1: Quantification of cellular metabolic activity. Figure S2: Gap closure analysis at 20 and 40 μM AH23848. Figure S3: Investigation of PGE2 release and COX-2 regulation in CAOV3 cells. Original western blot images are included in the supplementary file. Figure S4: Figure 2 Original western blot images. Figure S5: Figure 5 Original western blot images. Figure S6: Figure S3 Original western blot images.
