# Supplementary material for: Photodynamic Priming Improves the Anti-Migratory Activity of Prostaglandin E Receptor 4 Antagonist in Cancer Cells In Vitro
Source: Cancers (Basel). 2021 Oct 20;13(21):5259. doi: 10.3390/cancers13215259 (PMC8582354; doi:10.3390/cancers13215259)
Supplement: Supplementary file 1 [file cancers-13-05259-s001.zip › cancers-1388556-Supplementary.pdf]

cancers

MDPI

Supplementary Materials

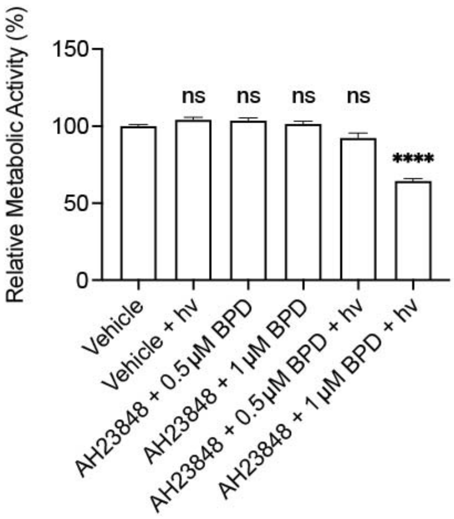

Figure S1. Quantification of cellular metabolic activity. OVCAR-5 cells were treated for 24 hours with the indicated agents (AH23848 dose was fixed at 40 µM), then the light activated groups received 690 nm light (0.1 J/cm², 10 mW/cm²). Metabolic activity was quantified 24 hours later using the MTT assay. Data is normalized to the vehicle (DMSO) control, then statistical analysis was performed using a one-way ANOVA and post hoc Tukey's test. Error bars represent the standard error of the mean. ****$p \leq 0.0001$; ns: nonsignificant.

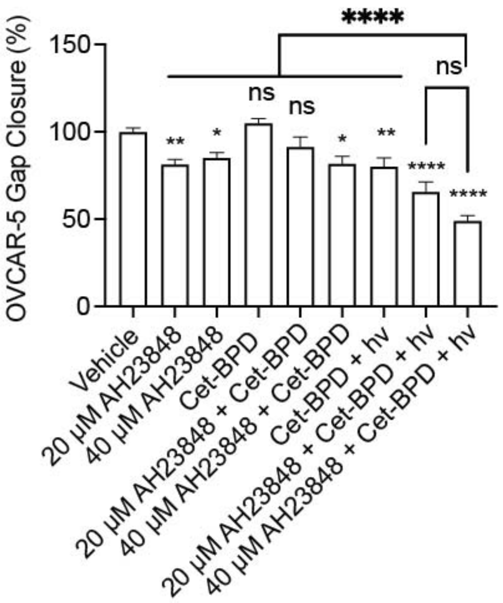

Figure S2. Gap closure analysis at 20 µM and 40 µM AH23848. OVCAR-5 cells were treated with the indicated agents for 24 hours, then light-activated at 690 nm (0.1 J/cm², 10 mW/cm²). Following irradiation, cells were scratched for gap closure analysis and normalized to the vehicle (DMSO) control. Statistical analysis was performed using a one-way ANOVA and post hoc Tukey's test. Error bars represent the standard error of the mean. *$p \leq 0.05$; **$p \leq 0.01$; ****$p \leq 0.0001$; ns: nonsignificant.

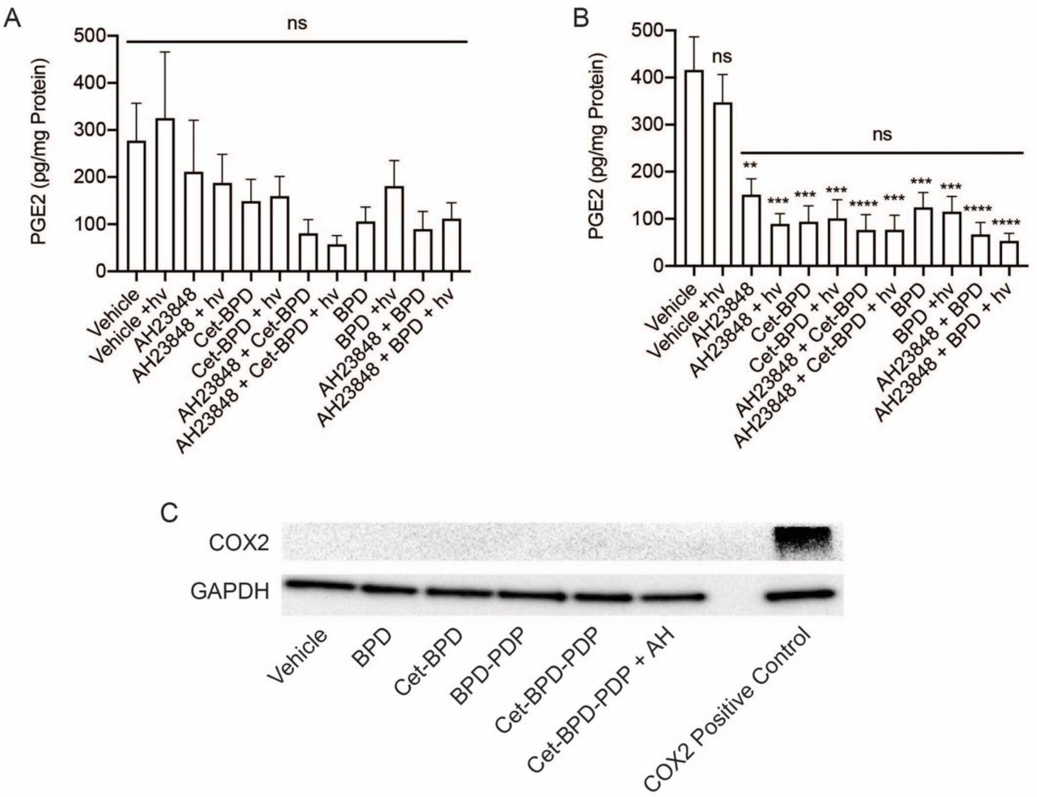

Figure S3. Investigation of PGE2 release and COX-2 regulation in CAOV3 cells. CAOV3 cells were treated with serum-free media supplemented with the indicated agents, and light-activated 24 hours later (0.1 J/cm^2, 10 mW/cm^2). Supernatants were collected at **(A)** 1 and **(B)** 4 hours post-PDP and assayed for PGE2 using the Prostaglandin E_2 ELISA Kit (514010, Cayman) **(C)** Lysates were collected at 24 hours post-PDP and probed for COX-2. GAPDH was included as a loading control. Statistical analysis was performed using a one-way ANOVA and post hoc Tukey's test. Error bars represent the standard error of the mean. ** $p \leq 0.01$; *** $p \leq 0.001$; **** $p \leq 0.0001$; ns: nonsignificant. Original western blot images (Supplementary Figure S6).

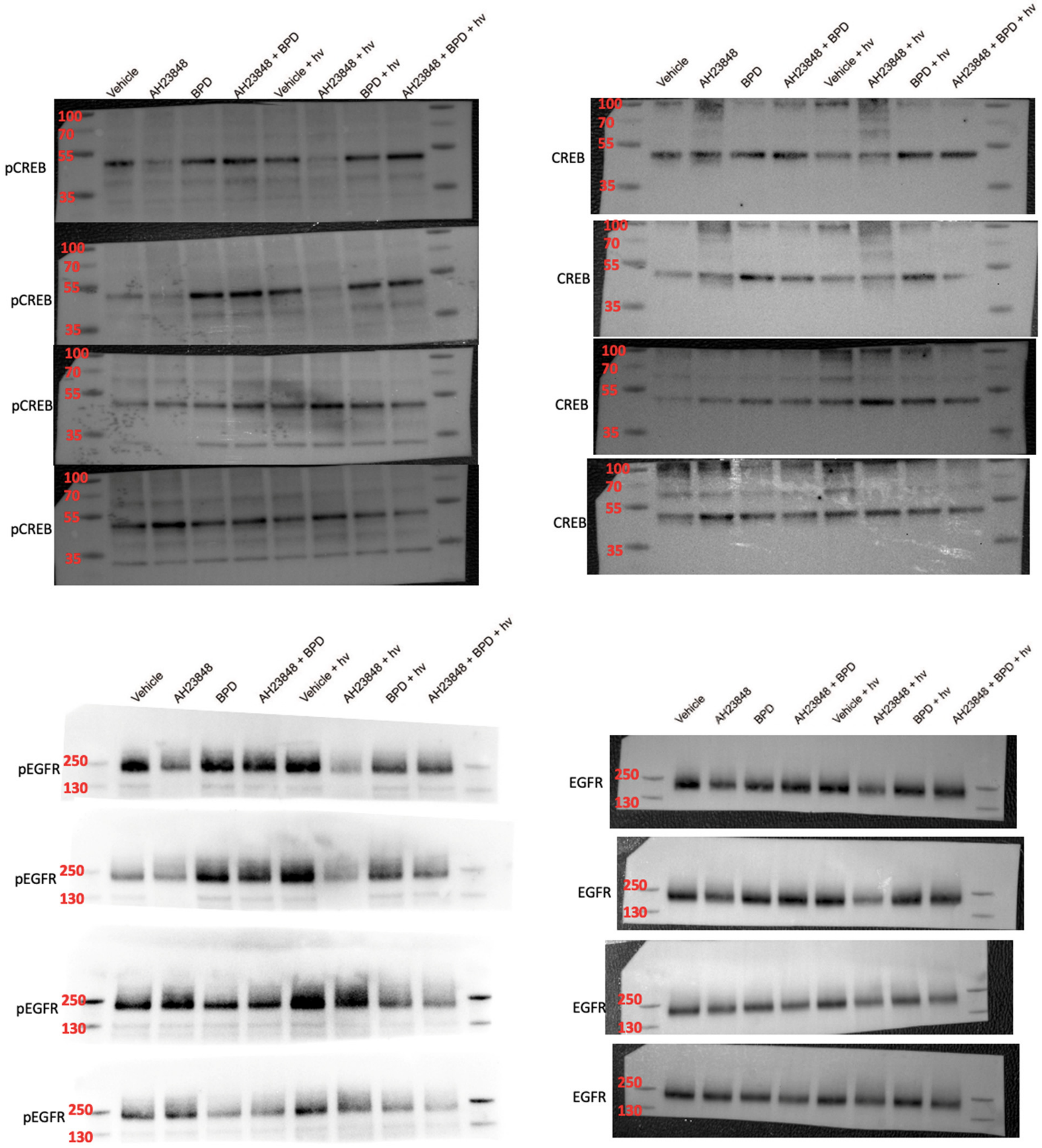
Vehicle
AH23848
BPD
AH23848 + BPD
Vehicle + hv
AH23848 + hv
BPD + hv
AH23848 + BPD + hv
pCREB
CREB
pEGFR
EGFR
100
70
55
35
250
130

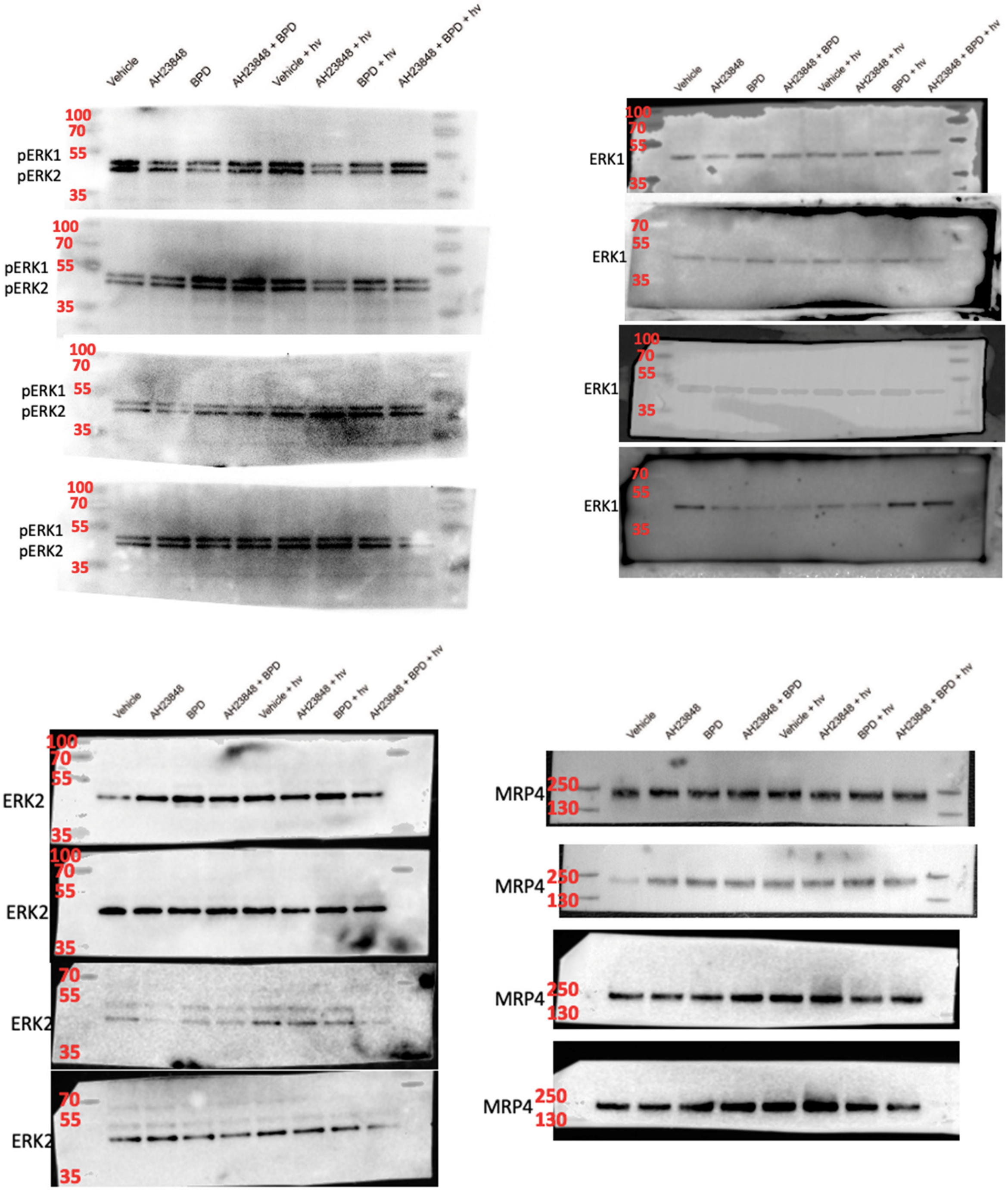
Vehicle
AH23848
BPD
AH23848 + BPD
Vehicle + hv
AH23848 + hv
BPD + hv
AH23848 + BPD + hv
pERK1
pERK2
ERK1
ERK2
MRP4
100
70
55
35
250
130

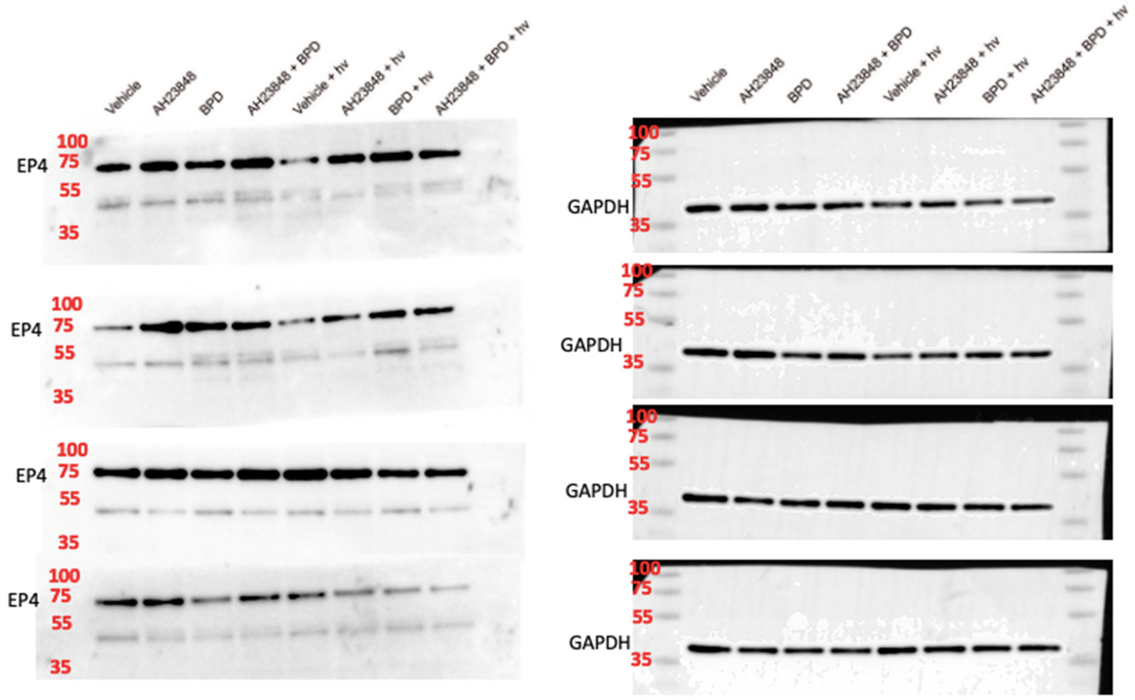

Figure S4. Figure 2 Original western blot images.

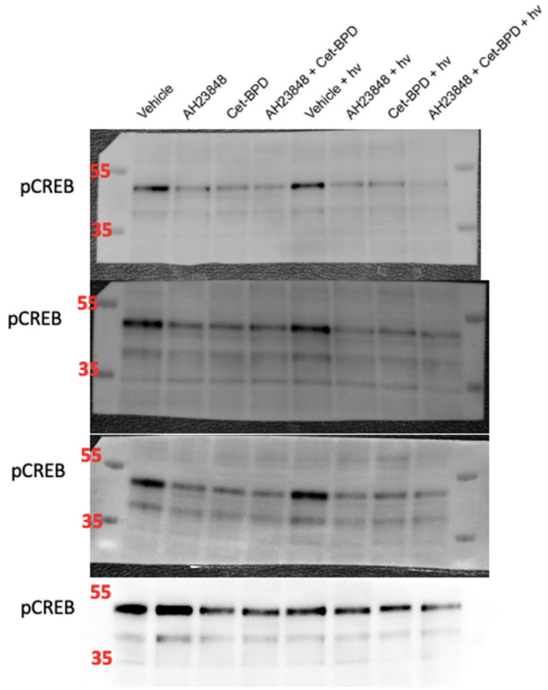
Vehicle
AH23848
Cet-BPD
AH23848 + Cet-BPD
Vehicle + hv
AH23848 + hv
Cet-BPD + hv
AH23848 + Cet-BPD + hv
pCREB
55
35

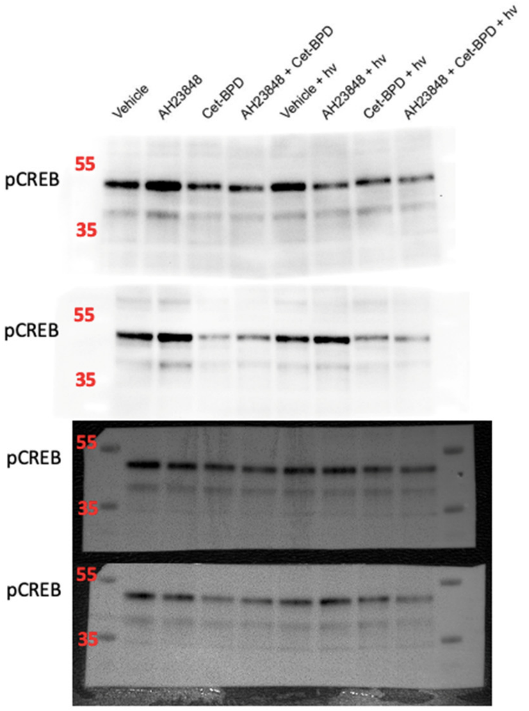
Vehicle
AH23848
Cet-BPD
AH23848 + Cet-BPD
Vehicle + hv
AH23848 + hv
Cet-BPD + hv
AH23848 + Cet-BPD + hv
pCREB
55
35

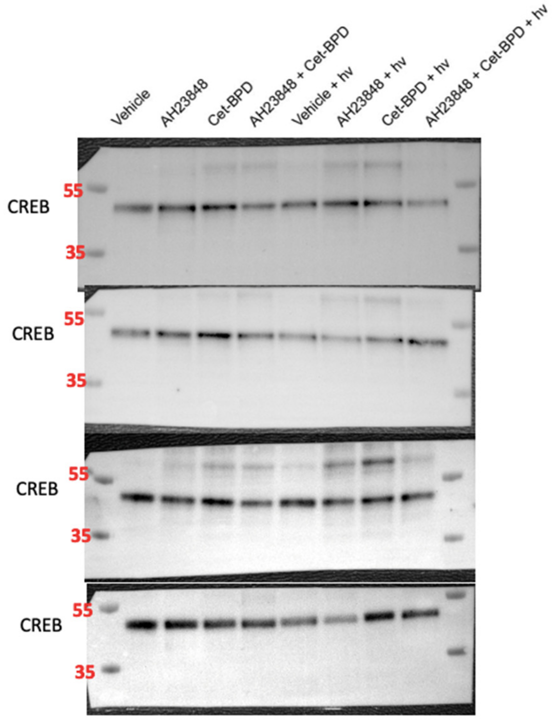
Vehicle
AH23848
Cet-BPD
AH23848 + Cet-BPD
Vehicle + hv
AH23848 + hv
Cet-BPD + hv
AH23848 + Cet-BPD + hv
CREB
55
35

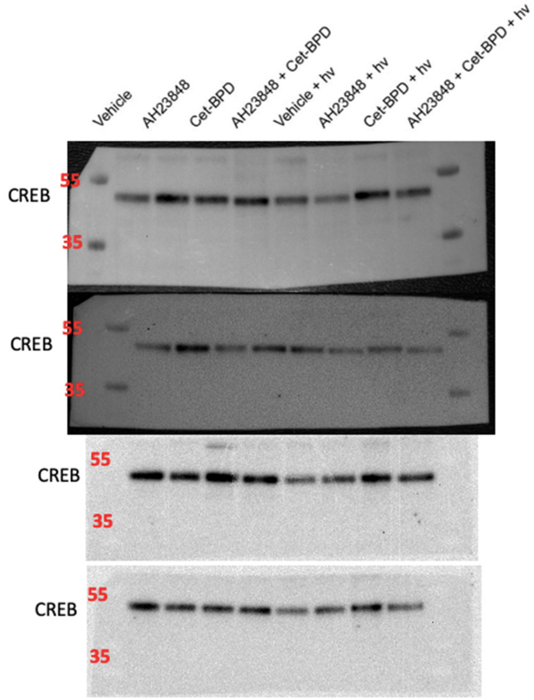
Vehicle
AH23848
Cet-BPD
AH23848 + Cet-BPD
Vehicle + hv
AH23848 + hv
Cet-BPD + hv
AH23848 + Cet-BPD + hv
CREB
55
35

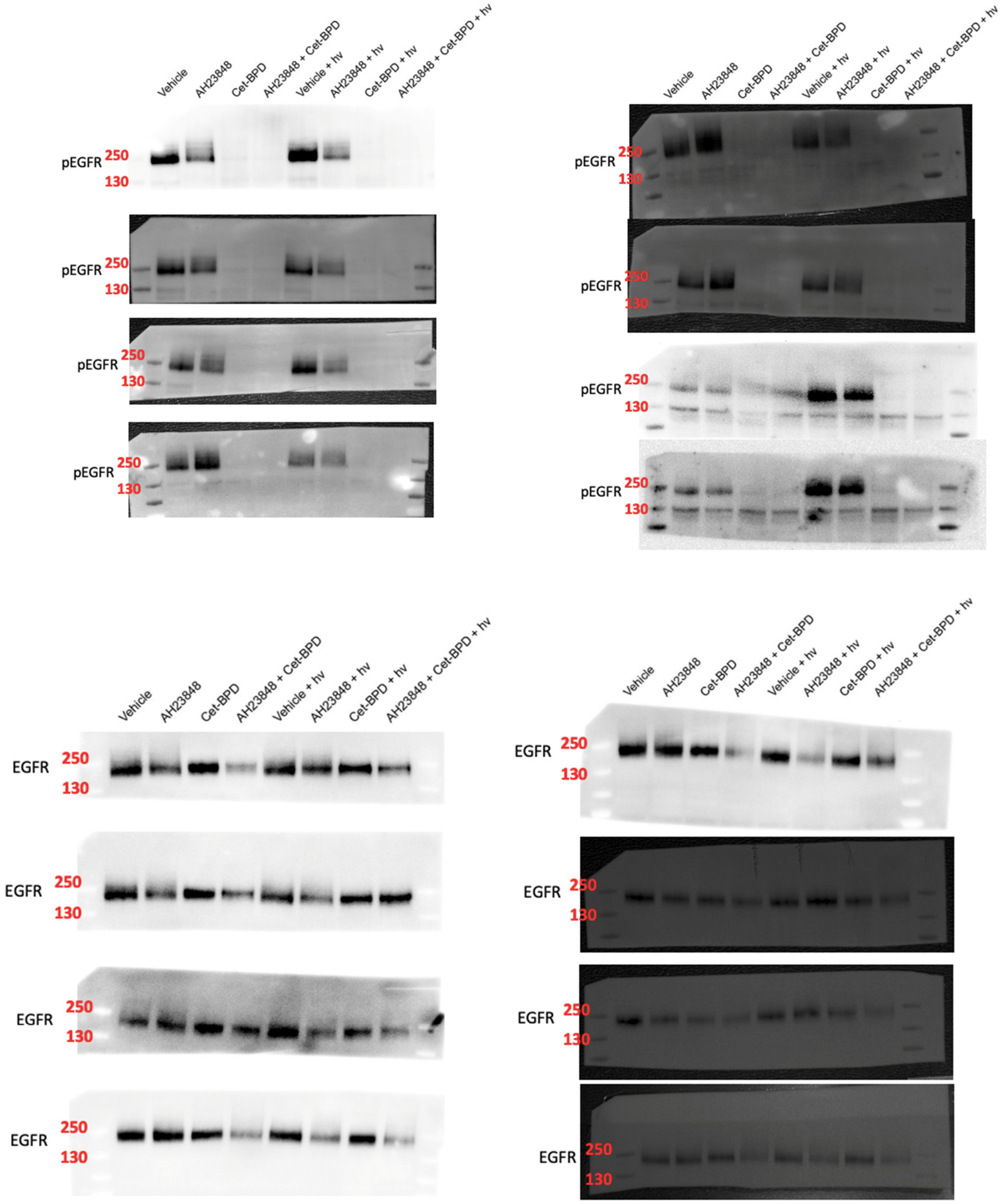
Vehicle
AH23848
Cet-BPD
AH23848 + Cet-BPD
Vehicle + hv
AH23848 + hv
Cet-BPD + hv
AH23848 + Cet-BPD + hv
pEGFR
250
130
EGFR

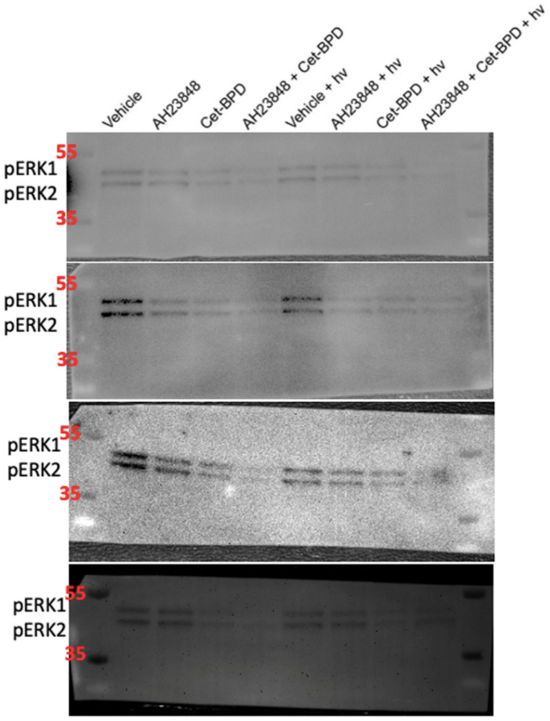
Vehicle
AH23848
Cet-BPD
AH23848 + Cet-BPD
Vehicle + hv
AH23848 + hv
Cet-BPD + hv
AH23848 + Cet-BPD + hv
pERK1
pERK2
55
35

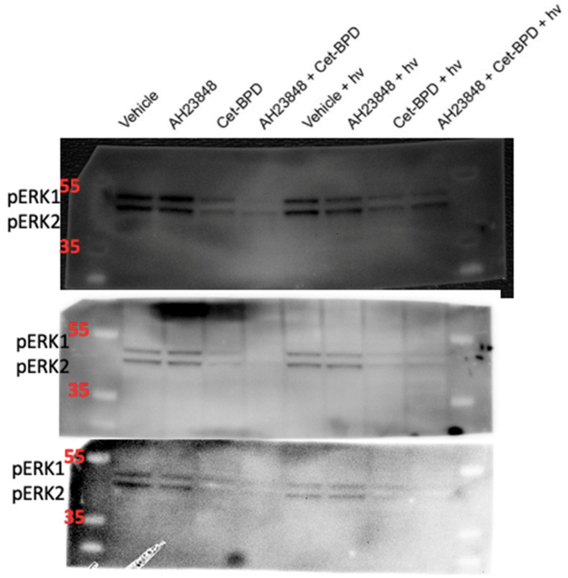
Vehicle
AH23848
Cet-BPD
AH23848 + Cet-BPD
Vehicle + hv
AH23848 + hv
Cet-BPD + hv
AH23848 + Cet-BPD + hv
pERK1
pERK2
55
35

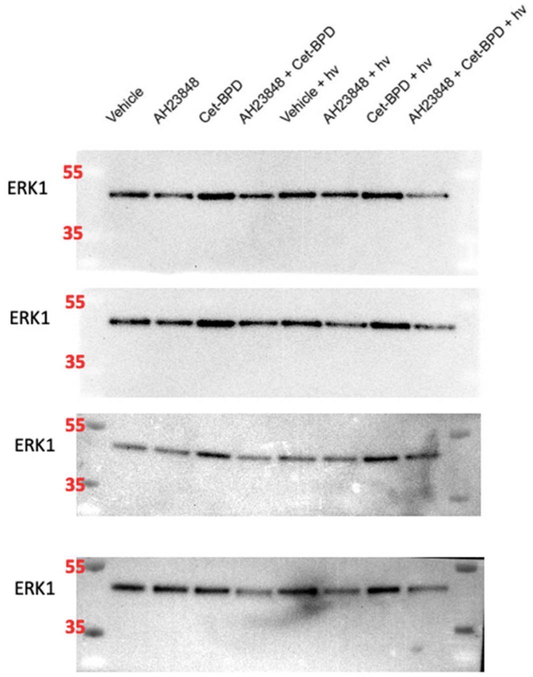
Vehicle
AH23848
Cet-BPD
AH23848 + Cet-BPD
Vehicle + hv
AH23848 + hv
Cet-BPD + hv
AH23848 + Cet-BPD + hv
ERK1
55
35

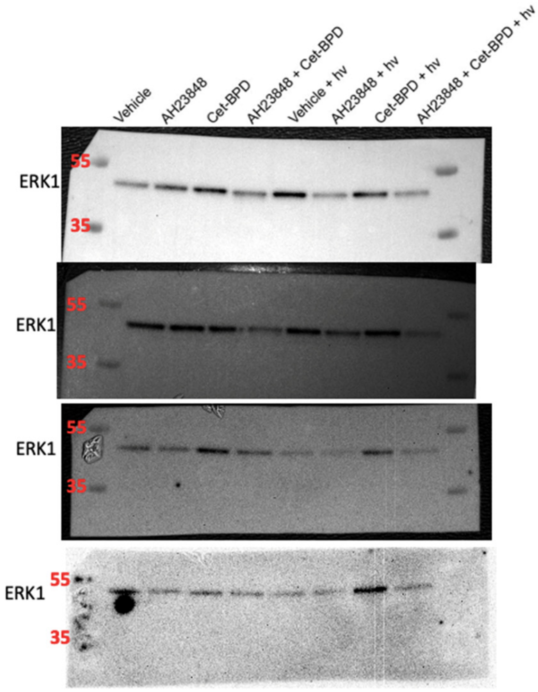
Vehicle
AH23848
Cet-BPD
AH23848 + Cet-BPD
Vehicle + hv
AH23848 + hv
Cet-BPD + hv
AH23848 + Cet-BPD + hv
ERK1
55
35

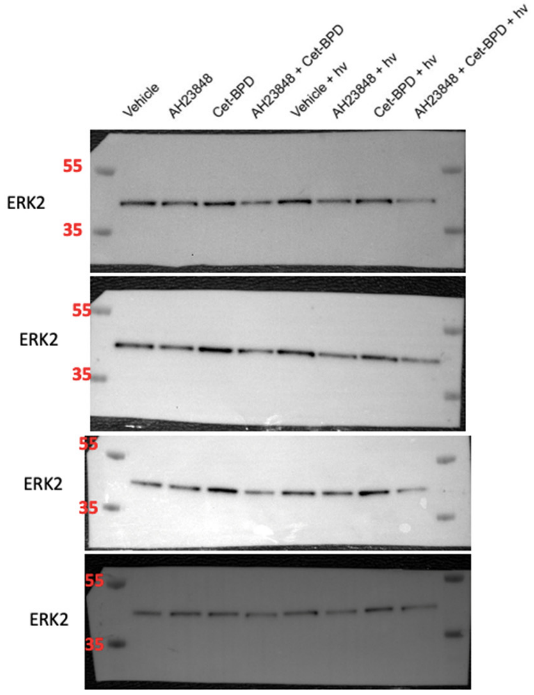
Vehicle
AH23848
Cet-BPD
AH23848 + Cet-BPD
Vehicle + hv
AH23848 + hv
Cet-BPD + hv
AH23848 + Cet-BPD + hv
ERK2
55
35

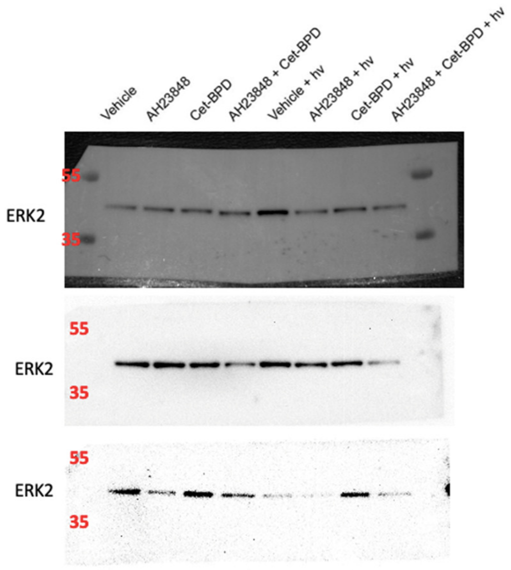
Vehicle
AH23848
Cet-BPD
AH23848 + Cet-BPD
Vehicle + hv
AH23848 + hv
Cet-BPD + hv
AH23848 + Cet-BPD + hv
ERK2
55
35

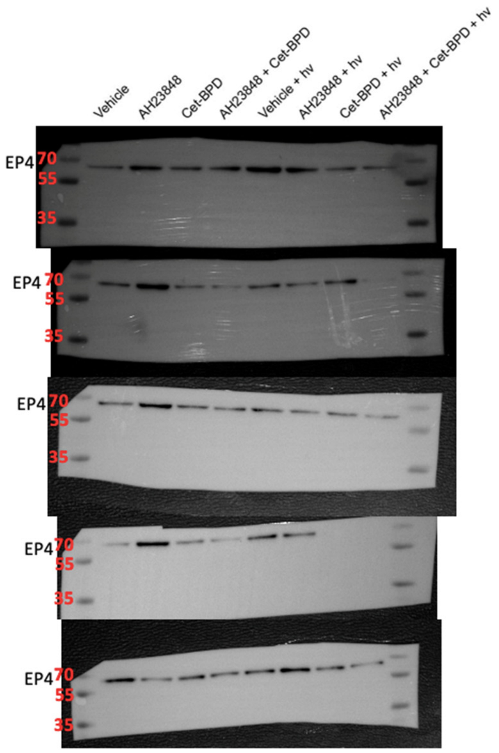
Vehicle
AH23848
Cet-BPD
AH23848 + Cet-BPD
Vehicle + hv
AH23848 + hv
Cet-BPD + hv
AH23848 + Cet-BPD + hv
EP4
70
55
35

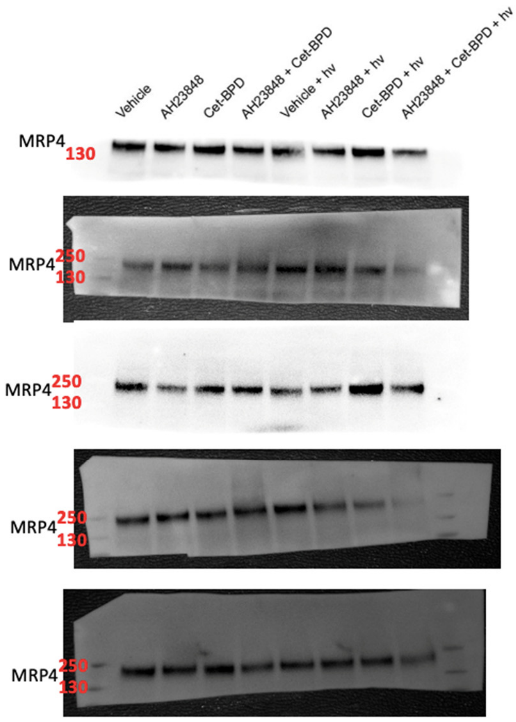
Vehicle
AH23848
Cet-BPD
AH23848 + Cet-BPD
Vehicle + hv
AH23848 + hv
Cet-BPD + hv
AH23848 + Cet-BPD + hv
MRP4
250
130

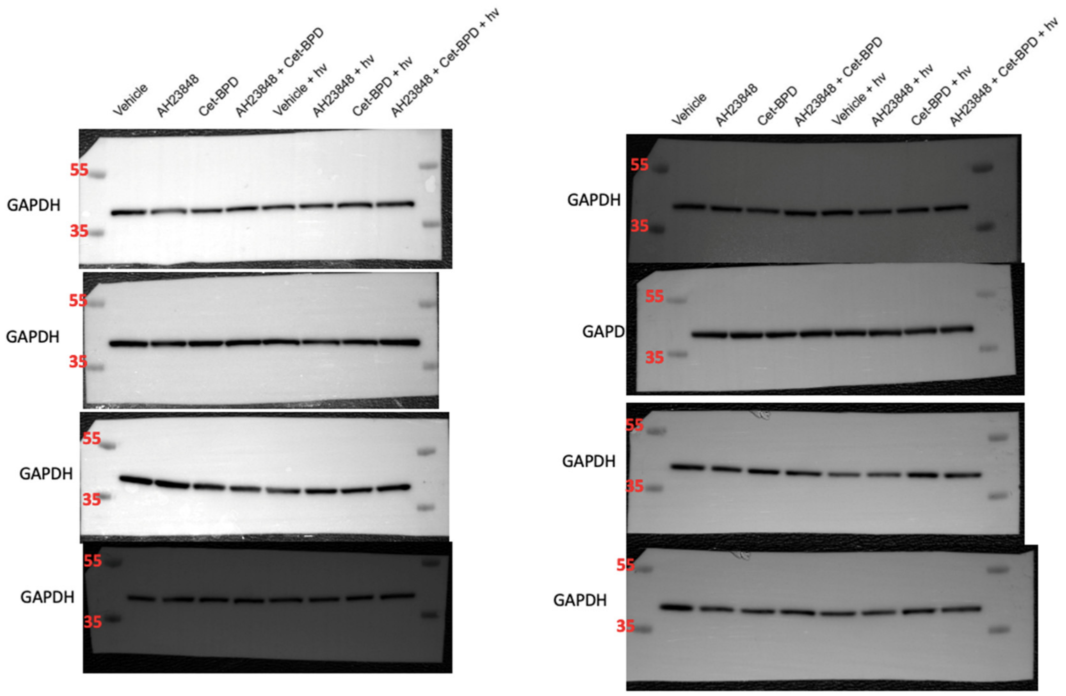

Figure S5. Figure 5 Original western blot images.

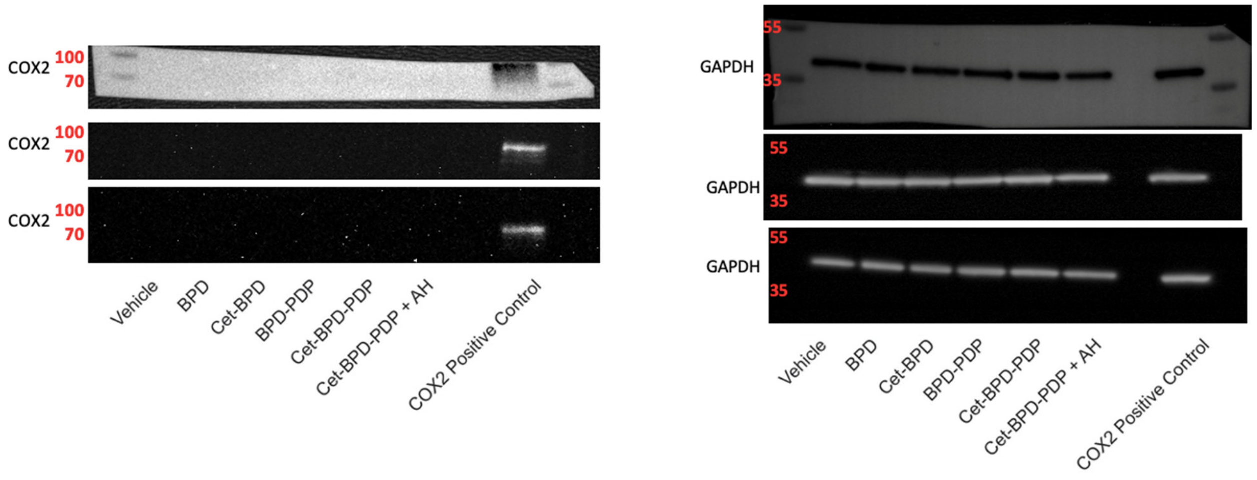

Figure S6. Figure S3 Original western blot images.
